# A multicenter, randomized, open-labelled, non-inferiority trial of sustained-release sarpogrelate versus clopidogrel after femoropopliteal artery intervention

**DOI:** 10.1038/s41598-023-29006-z

**Published:** 2023-02-13

**Authors:** Ahram Han, Taeseung Lee, Joongyub Lee, Suk-Won Song, Sang-Su Lee, In Mok Jung, Jin Mo Kang, Jun Gyo Gwon, Woo-Sung Yun, Yong-Pil Cho, Hyunmin Ko, Yang-Jin Park, Seung-Kee Min

**Affiliations:** 1 Division of Vascular Surgery, Department of Surgery, Seoul National University Hospital, 101 Daehak-Ro, Jongno-Gu, Seoul, 03080 Korea; 2 Seoul National University College of Medicine, Seoul, South Korea; 3 Division of Vascular Surgery, Bundang Seoul National University Hospital, Seongnam, South Korea; 4 Department of Preventive Medicine, Seoul National University College of Medicine, Seoul, South Korea; 5 Department of Cardiovascular Surgery, Gangnam Severance Hospital, Yonsei University College of Medicine, Seoul, South Korea; 6 Division of Vascular and Endovascular Surgery, Department of Surgery, School of Medicine, Pusan National University, Pusan National University Yangsan Hospital, Yangsan, South Korea; 7 Department of Surgery, Seoul Metropolitan Government–Seoul National University Boramae Medical Center, Seoul, South Korea; 8 Department of Surgery, Gil Hospital, Gachon University of Medicine and Science, Incheon, South Korea; 9 Department of Surgery, Korea University Hospital, Seoul, South Korea; 10 Department of Surgery, Yeungnam University Medical Center, Daegu, South Korea; 11 Division of Vascular Surgery, Department of Surgery, Asan Medical Center, Seoul, South Korea; 12 Department of Surgery, College of Medicine, Kyung Hee University, Seoul, South Korea; 13 Division of Vascular Surgery, Department of Surgery, Samsung Medical Center, Sungkyunkwan University School of Medicine, 81 Irwon-Ro, Gangnam-Gu, Seoul, 06351 South Korea

**Keywords:** Outcomes research, Cardiovascular diseases

## Abstract

Optimal antiplatelet therapy after endovascular therapy (EVT) for peripheral artery disease is controversial. This trial aimed to evaluate whether sarpogrelate plus aspirin was non-inferior for preventing early restenosis after femoropopliteal (FP) EVT compared to clopidogrel plus aspirin. In this open-label, prospective randomized trial, 272 patients were enrolled after successful EVT for FP lesions. Patients in each group received aspirin 100 mg and clopidogrel 75 mg or sarpogrelate 300 mg orally once per day for 6 months. The primary outcome was target lesion restenosis at 6 months, tested for noninferiority. Patient characteristics and EVT patterns were similar, except for increased inflow procedures in the sarpogrelate group and increased outflow procedures in the clopidogrel group. The sarpogrelate group showed a tendency of less restenosis at 6 months than the clopidogrel group (13.0% vs. 19.1%, difference 6.1 percentage points, 95% CI for noninferiority − 0.047 to 0.169). Secondary endpoints related to safety outcomes were rare in both groups. Risks of target lesion restenosis of the two intervention arm were uniform across most major subgroups except for those with coronary artery disease. In conclusion, Sarpogrelate plus aspirin is non-inferior to clopidogrel plus aspirin in preventing early restenosis after FP EVT. Larger multi-ethnic trials are required to generalize these findings.

*Trial registration*: National Institutes of Health Clinical Trials Registry (ClinicalTrials.gov identifier: NCT02959606; 09/11/2016).

## Introduction

Globally, more than 200 million people suffer from peripheral artery disease (PAD), and its prevalence is rising with the increasing aging population^1^. Symptomatic PAD presents as intermittent claudication, rest pain, or tissue loss and thus directly affects patients’ daily activity and diminishes quality of life^2–4^. The disease is additionally known to be associated with an increased risk of future cardiovascular events and death^5,6^. Therefore, medical therapy for PAD should aim to both reduce PAD symptoms and prevent subsequent cardiovascular events.

The role of medical therapy after revascularization is especially essential for symptomatic PAD patients who have undergone revascularization either by endovascular intervention or open surgery. According to a recent analysis of administrative data from the United States, there has been a rapid increase in major adverse limb events (MALE) during the first year after PAD revascularization, with 12.9% of patients readministered for MALE at a median of 4 months post-procedure^7^. To mitigate such increased risks of vascular events, current guidelines recommend intensified antithrombotic therapy after revascularization. However, even in the established guidelines, the post-revascularization drug regimens are not clear, nor are their duration, due to limited high-quality data. The 2016 American Heart Association/American College of Cardiology (ACC/AHA)^8^ and the 2017 European Society of Cardiology (ESC)/European Society for Vascular Surgery (ESVS) guidelines^9^ recommend aspirin and clopidogrel for 1–12 months, with a minimum of 1 month after endovascular revascularization (IIb and II recommendation, respectively) based on three small controlled trials. In the real world, the prescription patterns of antithrombotic medication are highly varied, with about a quarter of patients administered one or no antiplatelet drugs at discharge after endovascular revascularization^10^. This partially reflects the complexity of the PAD patient pool, which includes a significant portion at risk for bleeding, further complicating antithrombotic therapy.

Sarpogrelate is a selective 5-hydroxytryptamine (5-HT) 2A receptor antagonist that inhibits 5-HT-induced platelet aggregation^11^. Unlike other antiplatelets, sarpogrelate is unique in its ability to inhibit vasoconstriction and vascular smooth muscle cell (VSMC) proliferation through its interaction with 5-HT 2A receptors in the VSMC^11,12^. While its beneficial effects for preventing atherosclerotic disease progression and in-stent restenosis in coronary beds^13,14^ and effectiveness in relieving symptoms in patients with claudication^15^ have been demonstrated, research on its efficacy in PAD patients after endovascular revascularization is limited. Recently, a new sustained-release (SR) sarpogrelate has been made available, offering better patient compliance with a once-daily regimen compared to the thrice-daily dosing of the previous formulation.

Therefore, in the current SAFE (Sarpogrelate Anplone in Femoropopliteal artery intervention Efficacy) study we performed a clinical trial to assess the efficacy and safety of dual antiplatlet therapy (DAPT) with sarpogrelate and aspirin compared to clopidogrel and aspirin in PAD patients during the early periods following endovascular revascularization.

## Methods

### Study design

The detailed design of this trial has been published previously^16^. Briefly, the SAFE trial was a multicenter open-label prospective trial that randomized patients with successful endovascular femoropopliteal (FP) revascularization from 10 sites in Korea. The study’s objective was to assess the non-inferiority of DAPT with sarpogrelate and aspirin compared to clopidogrel and aspirin at 6 months after FP intervention. Informed consent was obtained from all subjects. The study was performed in compliance with the declaration of Helsinki, and the study protocol was approved by the Institutional Review Boards of all 10 participating institutions: Seoul national university hospital, Bundang Seoul national university hospital, Gangnam Severance hospital, Pusan national university Yangsan hospital, Seoul metropolitan government–Seoul national university Boramae medical center, Gachon university Gil hospital, Korea university hospital, Yeungnam university medical center, Asan medical center, and Samsung medical center.

### Study population

Eligible patients were adults with a significant atherosclerotic steno-occlusive lesion (≥ 50% diameter stenosis on angiography) of the FP artery. Patients were eligible for randomization after successful endovascular FP intervention (defined as < 30% residual stenosis on completion angiography) with adequate inflow and patent outflow. To achieve an adequate inflow and at least one patent below-the-knee (BTK) outflow, concomitant iliac procedures, and BTK procedures were permitted. Key exclusion criteria were known bleeding tendency, acute limb ischemia, inflammatory arterial disease, previous FP bypass or intervention on the same leg, or ongoing anticoagulation therapy. The inclusion and exclusion criteria are listed in the study protocol^16^.

### Randomization and study treatment

**Figure 1:**
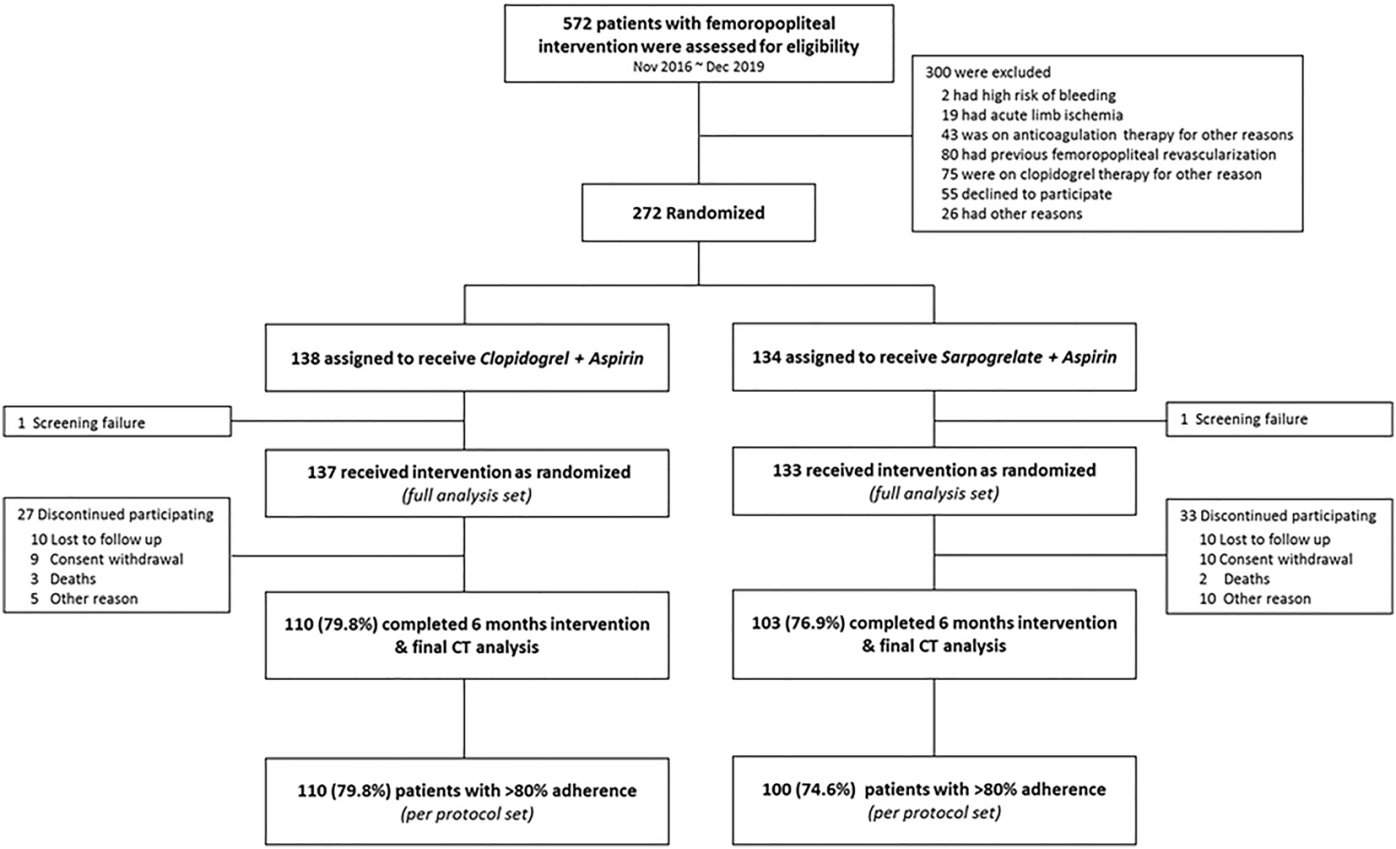
Consort diagram of the SAFE trial.

The study scheme is shown in Fig. 1. Patients were screened for eligibility before the revascularization procedure. Eligible patients received a loading dose of aspirin 100 mg and clopidogrel 300 mg on the day of the revascularization procedure unless taken previously. Patients were enrolled and randomized after the procedural success was demonstrated on completion angiography. Patients were randomized 1:1 to either the Clopidogrel group with clopidogrel 75 mg and aspirin 100 mg once daily or the Sarpogrelate group with SR sarpogrelate 300 mg and aspirin 100 mg once daily. Both groups received the study medication for 6 months. Patients were evaluated at 2 weeks, 3 months, and 6 months during outpatient visits. In addition, all patients were scheduled for follow-up CT angiography (CTA) at 6 months.

Randomization was conducted by an centralized, independent statistical core at the Medical Research Collaborating Center (MRCC, https://mrcc.snuh.org) of Seoul National University Hospital via a web-based randomization system. Permuted block randomization was used with blocks of sizes 4 or 6 to ensure a balance between the two treatment groups. Randomizations were stratified by lesion type (stenotic vs occlusive), use of stent, TASC classification (A/B vs C/D) and presence of critical limb ischemia. After patient enrollment by the investigator, the site study nurse logged into the interactive web-responsive system of the MRCC and entered details of the inclusion and exclusion criteria. Upon verification of the eligibility criteria, the randomization was performed according to the random allocation table of the MRCC, and the allocation results were delivered through the web-based randomization system. The randomization table was kept within the MRCC, and the information was inaccessible to the investigators until the last study visit of the last participant. The MRCC had no other role in the trial process.

### End points

The primary endpoint was the binary restenosis rate, defined as > 50% luminal reduction of the initially treated FP lesion on CTA or catheter angiography at six months post-intervention. Secondary endpoints were target lesion revascularization, major amputation (unplanned ipsilateral below-knee or above-knee amputation), major bleeding (bleeding requiring ≥ 2 units of blood transfusion, surgical intervention, or inotropic support), death from any cause, and serious adverse events during the study period^16^. Additionally, we also sought to identify risk factors of restenosis after FP revascularization.

### Sample size calculation

The sample size calculation was based on an assumed incidence of the primary endpoint (binary restenosis) of 11% in the clopidogrel group and 6% in the sarpogrelate group, according to the previous literature^17^. The non-inferiority margin was pre-defined clinically as 5%. With a power of 80%, one-sided α of 0.025, and assumed follow-up loss rate of 10%, 136 patients per group were required to demonstrate non-inferiority between the two groups.

### Statistical analysis

The main analysis of the primary endpoint was performed on the per-protocol set (PPS), consisting of all subjects that finished 6 months of follow-up and underwent a final 6-month assessment of ankle brachial index (ABI), CTA or angiography and showing > 80% of medication adherence. The non-inferiority hypothesis for the primary outcome was tested through a one-sided 97.5% confidence interval (CI) approach. The weighted difference of proportion of primary endpoint and the 95% CI around the difference between study treatments was calculated using a Wald test with continuity correction. The Z_CU_ method was used to calculate the p-value for non-inferiority^18^. If the lower bound of the 95% CI for the difference was above − 5 percentage points, non-inferiority was claimed. As a sensitivity analysis, the primary endpoint was also analyzed in the full analysis set (FAS) which of subjects had been randomized and received at least one dose of the study drug.

For other secondary outcomes, the proportion of subjects meeting the definition of endpoints was summarized using descriptive statistics in both FAS and PPS. The rates of major and minor bleeding and cardiovascular events were compared using the Chi-square test or Fisher exact test as appropriate. The rates of target lesion revascularization, all-cause mortality, and major amputation were compared between groups using log-rank tests and presented as survival curves constructed using Kaplan–Meier methods. Risk factors for the primary outcome were assessed using logistic regression models. Significance tests were two-sided for all analyses unless specified otherwise. A p-value of < 0.05 was considered a statistically significant difference between the groups. Statistical analysis was performed with SAS 9.4 (SAS institute Inc., Cary, NC, USA).

## Results

### Study participants

A total of 272 patients were enrolled and randomly assigned to receive aspirin plus sarpogrelate (n = 134, sarpogrelate group) or aspirin plus clopidogrel (n = 137, clopidogrel group) after successful endovascular therapy (EVT) for target FP lesion (Fig. 1). After randomization, two patients were excluded due to erroneous screening. Throughout the study, 19 patients withdrew their consent (10 in sarpogrelate group and nine in clopidogrel group), and 20 patients were lost to follow-up. Five patients died (two in the sarpogrelate group and three in the clopidogrel group) and 15 patients stopped participating due to other reasons (specifically, the addition of or change to other anticoagulant or antiplatelet, n = 10; medication discontinuation, n = 2; physician’s discretion, n = 3). Overall, 78.3% of the enrolled patients (213/272) underwent final imaging evaluation after 6 months of follow-up, and 77.2% (210/272) patients with > 80% adherence to the study drug were included in the analysis as the PPS.

### Baseline characteristics of patients

Baseline characteristics of the intention-to-treat population (i.e. the FAS) are shown in Table 1. The average patient age was 70.5 years and 13.6% of patients were female. There were no statistically significant differences in clinical characteristics between the two groups, with the exception of body mass index and estimated glomerular filtration rate (eGFR). The sarpogrelate group had a higher body mass index (24.0 ± 3.8 vs 23.0 ± 3.0 kg/m^2^) and lower eGFR (70.0 ± 29.9 vs 78.5 ± 33.1 mL/min) compared to the clopidogrel group (p = 0.01 and 0.03, respectively).

**Table 1:** Baseline patient characteristics. Data were compared using Chi-square test or t-test. SD, standard deviation; Hb A1c, hemoglobin A1C; eGFR, estimated glomerular filtration rate; ABI, ankle brachial index.

	Clopidogrel group (n = 137)	Sarpogrelate group (n = 133)	*p* value
Age, yr, mean ± SD	70.9 ± 8.6	70.0 ± 9.2	0.42
Male sex, n (%)	118 (86.1%)	115 (86.5%)	0.94
Body mass index, kg/m^2^, mean ± SD	23.0 ± 3.0	24.0 ± 3.8	0.011
Coexisting conditions, n (%)			
Current smoking	47 (34.3%)	46 (34.6%)	0.96
Hypertension	102 (74.5%)	97 (72.9%)	0.78
Hyperlipidemia	42 (30.7%)	42 (31.6%)	0.87
Diabetes mellitus	85 (62.0%)	81 (60.9%)	0.85
End stage renal disease	11 (8.0%)	14 (10.5%)	0.48
Known coronary artery disease	32 (23.4%)	29 (21.8%)	0.76
Known cerebrovascular disease	13 (9.5%)	15 (11.3%)	0.63
Medications, n (%)			
Statin therapy, n (%)	53 (38.7%)	56 (42.1%)	0.57
Antiplatelet therapy, n (%)	87 (63.5%)	83 (62.4%)	0.85
Laboratory values, mean ± SD			
Cholesterol, mg/dl	153.4 ± 43.1	154.8 ± 42.7	0.81
Hb A1c, %	7.6 ± 1.5	7.8 ± 1.3	0.50
eGFR, ml/min	78.5 ± 33.1	70.0 ± 29.9	0.026
Systolic blood pressure, mmhg	131.1 ± 16.43	132.44 ± 19.01	0.54
Rutherford category, n (%)			0.70
1	17 (12.4%)	16 (12.0%)	
2	38 (27.7%)	47 (35.3%)	
3	58 (42.3%)	47 (35.3%)	
4	7 (5.1%)	4 (3.0%)	
5	7 (5.1%)	8 (6.0%)	
6	10 (7.3%)	11 (8.3%)	
Baseline ABI, mean ± SD			
Left	0.74 ± 0.30	0.77 ± 0.28	0.41
Right	0.75 ± 0.30	0.74 ± 0.26	0.85

### Characteristics of endovascular therapy

Table 2 shows the details of the qualifying EVT performed for each target FP lesion. Approximately half of the included FP lesions were of TASC B. Among 270 intention-to-treat patients, 134 (49.6%) were treated with balloon angioplasty alone, while 136 (50.4%) needed either bare-metal stent (n = 73, 27.0%) or drug-eluting stent (n = 63, 23.3%). The type and devices used for the EVT were well-balanced between the two groups except for stent diameter (clopidogrel group 6.1 ± 0.7 mm; sarpogrelate group, 5.8 ± 0.7 mm; p = 0.03). While the TASC grade of co-existing aortoiliac lesion did not differ between the two groups, the sarpogrelate group had more concomitant inflow procedures performed during the initial EVT (19.6% vs 11.0%), whereas the clopidogrel group had more outflow procedures performed (21.2% vs 11.3%).

**Table 2:** Characteristics of the target lesions and endovascular therapy. Data were compared using Chi-square test, t-test or Fisher's exact test. * percentages calculated from patients with stent insertion. † percentage calculated from patients with balloon angioplasty. BMS, bare metal stent; BTK, below-the-knee; DCB, drug-coated balloon; DES, drug-eluting stent; EVT, endovascular therapy; FP, femoropopliteal; SD, standard deviation; TASC, trans-atlantic inter-society consensus.

	Clopidogrel group (n = 137)	Sarpogrelate group (n = 133)	*p* value
TASC II classification of target FP lesion, n (%)			
A	20 (14.6%)	31 (23.3%)	0.22
B	76 (55.5%)	64 (48.1%)	
C	26 (19.0%)	28 (21.1%)	
D	15 (11.0%)	10 (7.5%)	
EVT of the target FP lesion, n (%)			0.46
Stent insertion	66 (48.2%)	70 (52.6%)	
* BMS*	38 (57.6%)*	35 (50%)*	
* DES*	28 (42.4%)*	35 (50%)*	
Balloon angioplasty only	71 (51.8%)	63 (47.0%)	
* Plain balloon*	8 (11.4%)†	4 (6.3%)†	
* DCB*	62 (88.6%)†	59 (93.7%)†	
FP EVT device size, mean ± SD			
Stent diameter, mm	6.09 ± 0.74	5.83 ± 0.66	0.031
Stent length, mm	14.3 ± 9.2	14.1 ± 10.7	0.93
Balloon diameter, mm	5.1 ± 0.8	5.2 ± 0.8	0.48
Co-existing aortoiliac lesion, n (%)			0.52
TASC A	36 (26.28%)	44 (33.08%)	
TASC B	26 (18.98%)	26 (19.55%)	
TASC C	11 (8.03%)	12 (9.02%)	
TASC D	1 (0.73%)	5 (3.76%)	
None			
Concomittant procedure, n (%)			0.026
Inflow	15 (11.0%)	26 (19.6%)	
Outflow	29 (21.2%)	15 (11.3%)	
None	93 (67.9%)	91 (68.4%)	
BTK runoff, n (%)			0.81
1	22 (16.1%)	25 (18.8%)	
2	33 (24.1%)	32 (24.1%)	
3	82 (59.9%)	76 (57.1%)	

**Figure 2:**
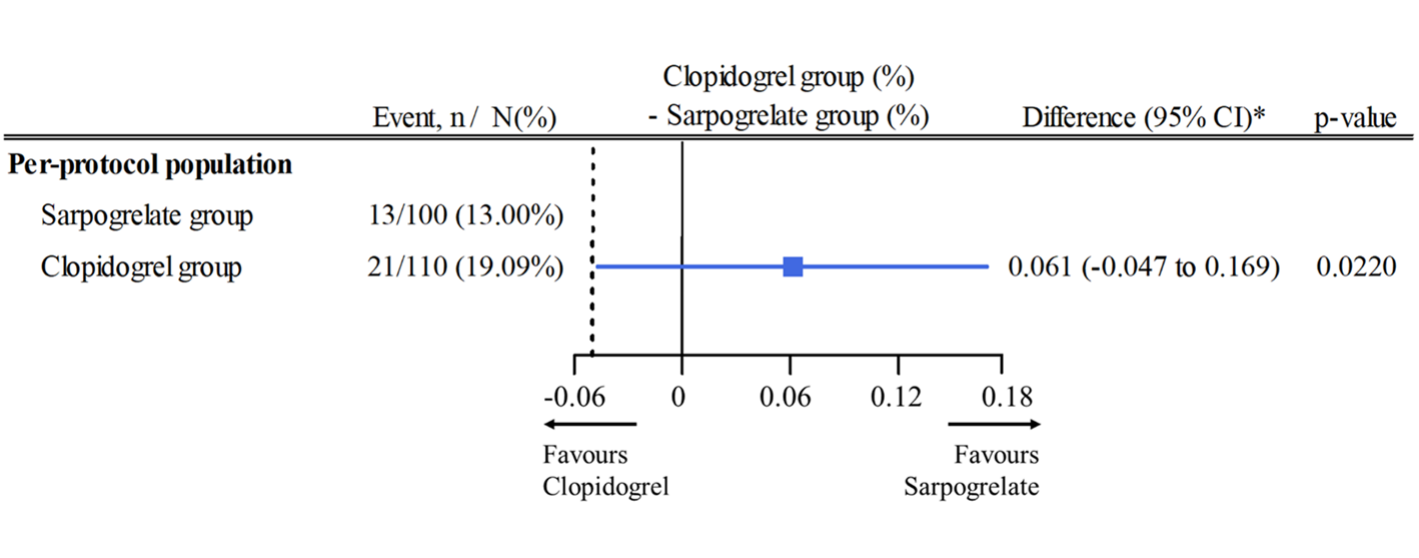
Noninferiority test for the primary outcome of target lesion restenosis.

The primary outcome of target lesion restenosis at 6 months was found in 19.1% (21/110) of the clopidogrel group and in 13% (13/100) of the sarpogrelate group (absolute risk difference 6.1%, 95% CI − 4.7 − 16.9% by Wald test with continuity correction). The non-inferiority of sarpogrelate compared to the clopidogrel was established, as the lower limit of the 95% CI was within the predefined margin of 0.05 (p-value for non-inferiority, 0.02; Fig. 2). However, when sensitivity analysis was performed in the FAS, the non-inferiority of sarpogrelate was not shown, despite similar risk reduction compared to the clopidogrel group (risk difference 5.4%, 95% CI, − 5.2 − 16.0%) (Figure S1).

**Figure 3:**
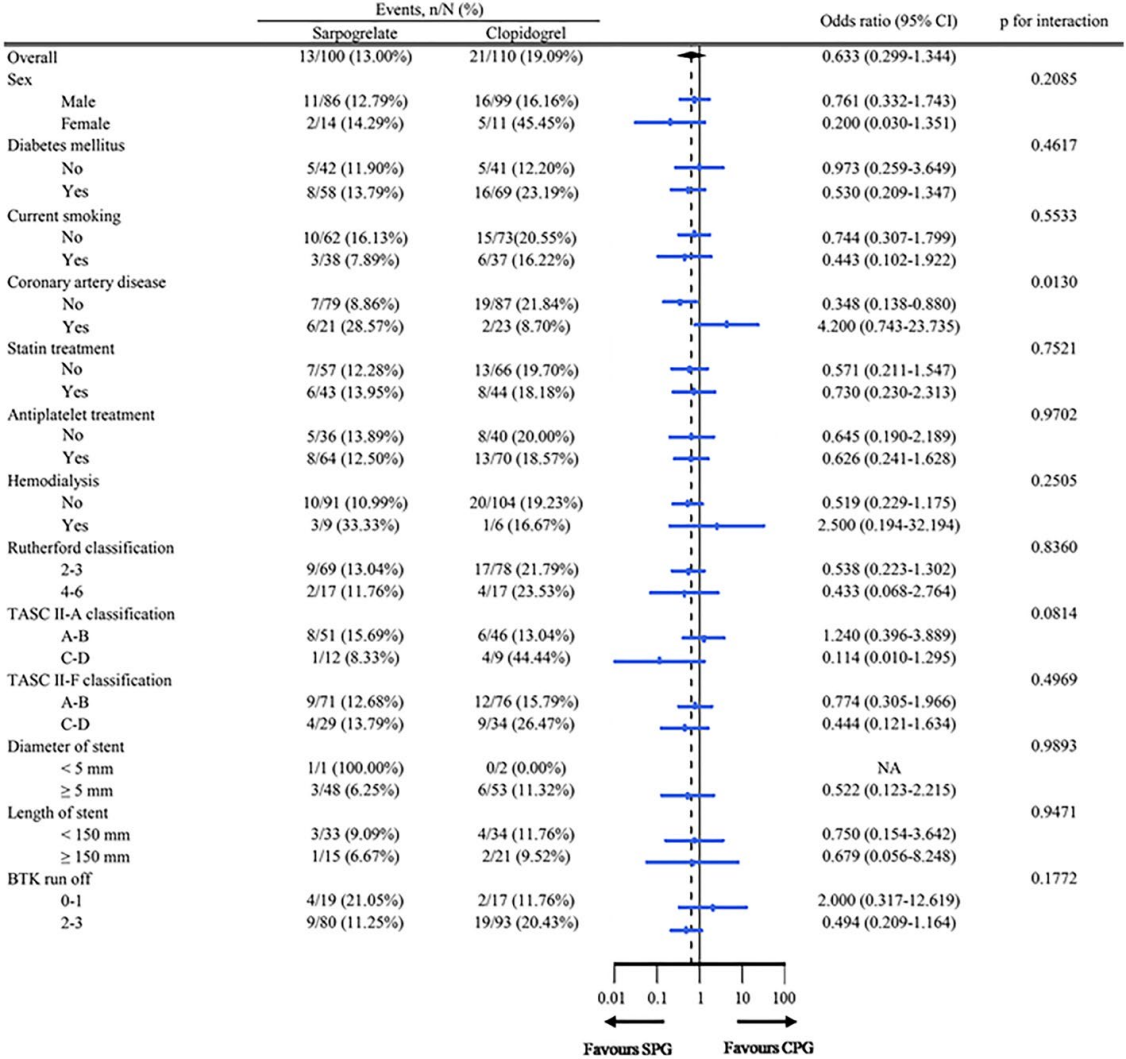
Subgroup analysis for the risk of target lesion restenosis.

Prespecified subgroup analysis revealed that risk of target lesion restenosis between the two intervention arms was similar across most major subgroups (Fig. 3). The only difference between the sarpogrelate and clopidogrel treatment was noted when the patients were grouped based on the presence of coronary artery disease (CAD); interestingly, those without coronary artery disease seemed to benefit more from sarpogrelate use (odds ratio [OR] 0.35, 95% CI 0.14 − 0.88), whereas those with coronary artery disease benefitted more from clopidogrel (OR 4.20, 95% CI 0.74 − 23.7).

### Secondary outcomes

**Figure 4:**
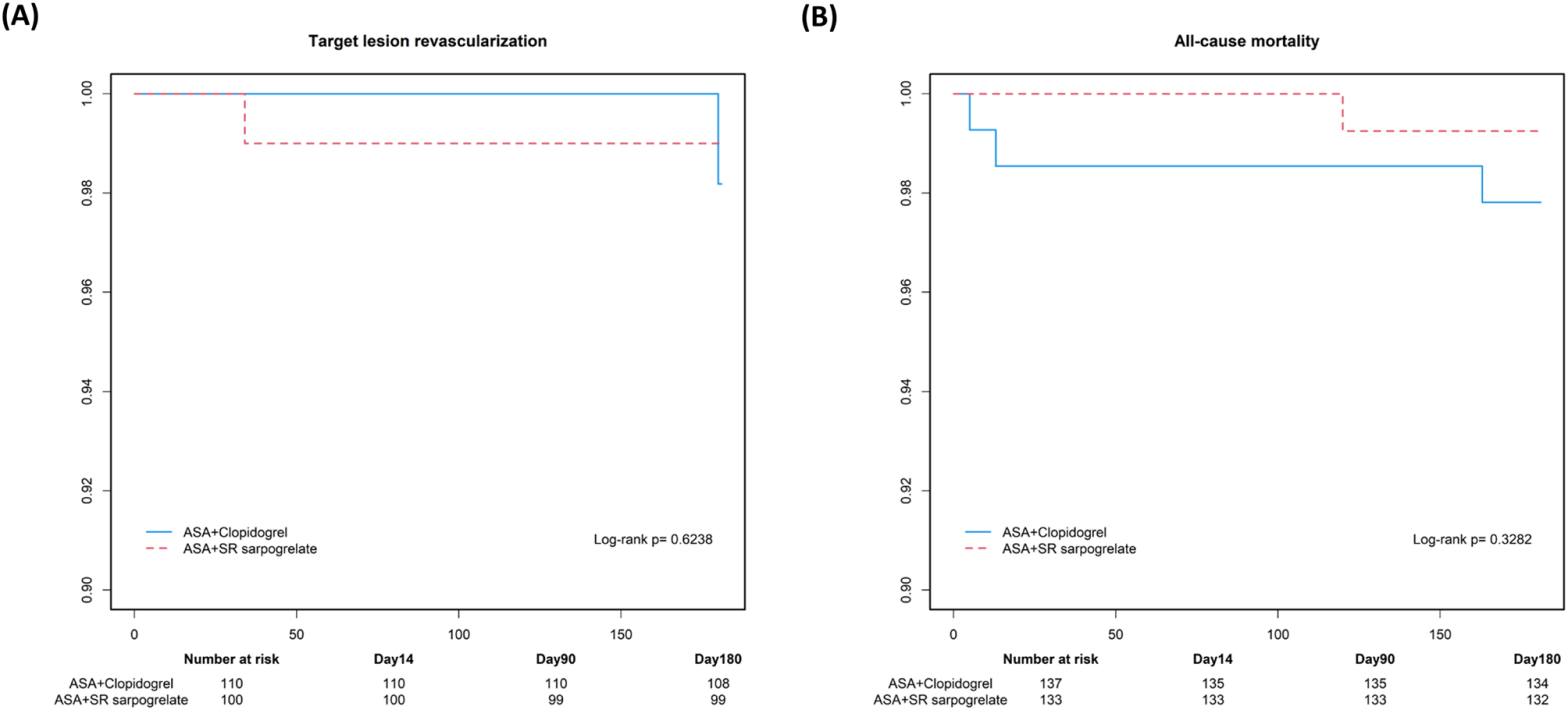
Kaplan–Meier analysis of the (**A**) target lesion revascularization and (**B**) all-cause mortality. There were no differences in the target lesion revascularization or all-cause mortality between the sarpogrelate group and the clopidogrel group according to the logistic regression analysis.

The occurrence of secondary outcome events of target lesion revascularization, major amputation, serious adverse event, major bleeding, mortality were overall rare, with no difference between the two groups (Table 3). Two patients in each group required revascularization of the target lesion (Fig. 4A), and one additional patient in the sarpogrelate group required EVT at a site separate from the initially treated vessel. Ischemic or thrombotic events in other vascular beds, including cardiac and cerebral, were also rare (one ischemic stroke took place in the clopidogrel group and three acute coronary syndromes took place in the sarpogrelate group). Regarding safety, there were numerically more bleeding complications in the clopidogrel group (two major, three minor) than in the sarpogrelate group (one minor), but the difference was not statistically significant (Table 3). All-cause mortality was also similar between the two groups (clopidogrel group, 2.2% vs sarpogrelate group 1.5%; Table 3, Fig. 4B.) The complete list of adverse events that took place during the study period is shown in Table S1.

**Table 3:** Primary and secondary outcome events. Data were compared using Chi-square test or Fisher's exact test. *1 cerebral hemorrhage, 1 hemoglobin drop needing transfusion in the clopidogrel group. †2 epistaxis, 1 upperarm petechiae in the clopidogrel group, 1 vitreous hemorrahge in the sarpogrelate group. ‡Causes of death were septic shock (n = 1), heart failure (n = 1), unknown (n = 1) in clopidogrel group, and myocardial infarction (n = 1), bowel perforation (n = 1) in the sarpogrelate group. § Any serious adverse event defined as all-cause death, ischemic stroke, transient ischemic attack, systemic embolism, acute coronary syndrome, early thrombotic occlusion, major bleeding, and major amputation.

	Clopidogrel group	Sarpogrelate group	*p* value
Primary outcome events – *per-protocol set*	*n* = *110*	*n* = *100*	
Target lesion restenosis, n (%)	21 (18.8%)	14 (13.3%)	0.28
Secondary outcome events – *full analysis set*	*n* = *137*	*n* = *133*	
Target lesion revascularization, n (%)	2 (1.5%)	2 (1.6%)	1.00
Target vessel revascularization, n (%)	0 (0%)	1 (0.8%)	0.49
Major amputation,n (%)	0 (0%)	0 (0%)	
Acute coronary syndrome, n (%)	0 (0%)	3 (2.4%)	0.12
Ischemic stroke, n (%)	1 (0.8%)	0 (0%)	1.00
Transient ischemic attack, n (%)	0 (0%)	0 (0%)	
Systemic embolism, n (%)	0 (0%)	0 (0%)	
Major bleeding complication, n (%)*	2 (1.5%)	0 (0%)	0.50
Minor bleeding complication, n (%)†	3 (2.3%)	1 (0.8%)	0.62
All-cause mortality, n (%)‡	3 (2.2%)	2 (1.5%)	1.00
Any serious adverse events, n (%)§	6 (4.4%)	5 (3.8%)	0.80

### Factors associated with target lesion restenosis

The treatment group was not associated with target lesion restenosis (Table S2). Significant factors in the multivariate analysis were TASC grade of the FP lesion (C/D vs A/B, adjusted OR 23.1, 95% CI 1.5–347.3, p = 0.02, TASC A/B as reference), and insertion of stents during initial EVT (adjusted OR 0.02, 95% CI 0.001–0.33; p < 0.01) (Table S2).

## Discussion

The current trial involving patients who had undergone successful FP EVT showed that the sarpogrelate plus aspirin (sarpogrelate group) is non-inferior to the clopidogrel plus aspirin (clopidogrel group) in terms of 6-month target lesion restenosis. The limb-related events and cardiovascular events of the two groups were also comparable.

After EVT for PAD, antiplatelet agents are used for three purposes, including prevention of early thrombosis, maintenance of long-term patency, and prevention of cardiovascular events. While aspirin- or clopidogrel-based monotherapy or DAPT is commonly used for these purposes^8^ subsequent risks of limb and cardiovascular events after EVT remain substantial^5,7^. Especially limb events, including revascularization and acute limb ischemia, constitute a major cause of hospitalization after EVT^7,19^.

The best antithrombotic treatment for FP intervention remains a matter of debate. The ACC/AHA^8^ and ESC/ESVS guidelines^9^ recommend aspirin and clopidogrel for 1–12 months following EVT. However, clopidogrel has several issues regarding PAD treatment, including the inability to prevent restenosis, increased bleeding complications when combined with aspirin^20^, and clopidogrel resistance^21^. Despite the drawbacks of the current medication after EVT, studies on alternative regimens have been limited. Three RCTs of small sample size have shown that cilostazol reduced angiographic restenosis after FP EVT^22–24^, and cilostazol was added as a possible alternative to clopidogrel in combination with aspirin in a recent guideline from the European Society of Vascular Medicine^25^. Recently, the VOYAGER PAD trial showed that low-dose rivaroxaban and aspirin reduced the risk of composite outcomes, including cardiovascular and limb events, in patients with lower extremity EVT compared to aspirin alone^26,27^. However, a direct comparison between the VOYAGER regimen and DAPT of aspirin plus clopidogrel is needed to prove its superiority or non-inferiority over the most commonly used regimen for post-EVT.

Sarpogrelate is an attractive drug choice for PAD considering its inhibitory effects on antiplatelet aggregation and vasoconstriction that prevent early thrombus and anti-vascular smooth muscle proliferation properties that prevent intimal hyperplasia^11,12^. In addition, by protecting endothelial cells dose-dependently and reducing the ICAM-1 level in a hyperglycemic state, sarpogrelate may also improve long-term patency after EVT in PAD patients with glucose intolerance^28^. Previously, Chen et al.^17^ reported that sarpogrelate plus aspirin is comparable to DAPT of clopidogrel plus aspirin after FP EVT based on small randomized trial of 120 patients. However the comparability was poorly supported as the trial which lacked details on the study design or statistical power, and merely showed a non-significant difference in stenosis recurrence between the two groups.

To the best of our knowledge, this study was the first to show the efficacy of SR sarpogrelate after PAD EVT. We enrolled 272 patients at 10 tertiary centers in Korea, which represents the current trend of EVT for FP disease in Korea. Further comparison with data from the Western population would highlight racial or ethnic differences in EVT for PAD. The sarpogrelate group showed a tendency of less restenosis at 6 months than the clopidogrel group (13% vs 19%) without statistically significant difference. Secondary endpoints of safety outcomes were very rare in both groups, showing that both DAPT regimens are safe after FP EVT.

Interestingly, in subgroup analysis, clopidogrel and aspirin showed better efficacy for reducing restenosis in CAD patients than sarpogrelate and aspirin together. A larger-scale study is needed to prove this concept. Multivariate analysis revealed TASC C/D lesions (vs A/B lesions) and only balloon angioplasty (vs stenting) were significant risk factors for restenosis at 6 months.

Another noteworthy point is that a sarpogrelate SR formulation requiring once-daily dosing rather than thrice-daily was used in our study. Multiple dosing is a well-known risk factor for medication nonadherence, especially in chronic diseases^29^. A SR sarpogrelate formula was recently developed and approved by the Korean FDA in 2015, simplifying the medication regimen for patients with multimorbidity.

This study has limitations. First, although a reasonable number of participants proved the non-inferiority of SR sarpogrelate to clopidogrel, this study was performed on a limited population in Korea. For generalization, multinational studies in a large population are needed. Second, the primary endpoint was restenosis at 6 months, and thus, only short-term follow-up data were available for this study. Post hoc data collection and 2-year analysis is anticipated and may reveal additional information. Third, the method of EVT was chosen at the discretion of the physician, and the study was not powered to compare the effectiveness of sarpogrelate and clopidogrel within each type of EVT (i.e. drug-coated balloons, drug-eluting stents, bare-metal stents, and plain balloon angioplasty). As in coronary beds, different medication regimens may prove more beneficial after different types of EVT. Fourth, although we had planned for a 10% dropout rate, only 78% of the randomized patients completed the six months intervention. This may have contributed to the insignificant results of the sensitivity analysis in the FAS analysis. In addition, a higher dropout rate in the per-protocol analysis could have resulted in a larger type I error than we expected, diluting the actual difference between the two intervention groups. Thus, larger-scale trials are warranted to confirm our results. Lastly, the noninferiority margin of our study was based on an expert panel discussion on the clinically meaningful difference in restenosis rate in FP lesions. While the selection of noninferiority margin needs to consider the standard treatment's effect estimate by pooling the relevant placebo-controlled randomized trial results, this was not possible as there was only one trial with inadequate sample size^30^.

In conclusion, results from the current trial suggest that sarpogrelate plus aspirin is non-inferior in preventing early restenosis after FP EVT compared to clopidogrel plus aspirin. Additional clinical trials in larger multinational, multiethnicity trial populations are warranted to generalize these findings.

### Supplementary Information

Supplementary Information.

## Data Availability

Individual data for the paper are available from the corresponding author on reasonable request.
