# Author Correction: A multicenter, randomized, open-labelled, non-inferiority trial of sustained-release sarpogrelate versus clopidogrel after femoropopliteal artery intervention

**DOI:** 10.1038/s41598-024-66740-4

**Published:** 2024-08-06

**Authors:** Ahram Han, Taeseung Lee, Joongyub Lee, Suk‑Won Song, Sang‑Su Lee, In Mok Jung, Jin Mo Kang, Jun Gyo Gwon, Woo‑Sung Yun, Yong‑Pil Cho, Hyunmin Ko, Yang‑Jin Park, Seung‑Kee Min

**Affiliations:** 1 Division of Vascular Surgery, Department of Surgery, Seoul National University Hospital, 101 Daehak-ro, Jongno-gu, Seoul, 03080 Korea; 2 Seoul National University College of Medicine, Seoul, South Korea; 3 Division of Vascular Surgery, Bundang Seoul National University Hospital, Seongnam, South Korea; 4 Department of Preventive Medicine, Seoul National University College of Medicine, Seoul, South Korea; 5 Department of Cardiovascular Surgery, Gangnam Severance Hospital, Yonsei University College of Medicine, Seoul, South Korea; 6 Division of Vascular and Endovascular Surgery, Department of Surgery, School of Medicine, Pusan National University, Pusan National University Yangsan Hospital, Yangsan, South Korea; 7 Department of Surgery, Seoul Metropolitan Government–Seoul National University Boramae Medical Center, Seoul, South Korea; 8 Department of Surgery, Gil Hospital, Gachon University of Medicine and Science, Incheon, South Korea; 9 Department of Surgery, Korea University Hospital, Seoul, South Korea; 10 Department of Surgery, Yeungnam University Medical Center, Daegu, South Korea; 11 Division of Vascular Surgery, Department of Surgery, Asan Medical Center, Seoul, South Korea; 12 Department of Surgery, College of Medicine, Kyung Hee University, Seoul, South Korea; 13 Division of Vascular Surgery, Department of Surgery, Samsung Medical Center, Sungkyunkwan University School of Medicine, 81 Irwon-ro, Gangnam-gu, Seoul, 06351 South Korea

Correction to: *Scientific Reports* 10.1038/s41598-023-29006-z, published online 13 February 2023

The original version of this Article contained an error in Affiliation 6, which was incorrectly given as ‘Division of Vascular and Endovascular Surgery, Department of Surgery, Yangsan Hospital, Pusan National University, Yangsan, South Korea’. The correct affiliation is listed below.

Division of Vascular and Endovascular Surgery, Department of Surgery, School of Medicine, Pusan National University, Pusan National University Yangsan Hospital, Yangsan, South Korea.

The original Article has been corrected.

