# Supplementary material for: A multicenter, randomized, open-labelled, non-inferiority trial of sustained-release sarpogrelate versus clopidogrel after femoropopliteal artery intervention
Source: Sci Rep. 2023 Feb 13;13:2502. doi: 10.1038/s41598-023-29006-z (PMC9925771; doi:10.1038/s41598-023-29006-z)

Supplemental material

**Table S1. All adverse event (according to MedDRA)**

| Full analysis set | Clopidogrel + Aspirin  (n=137) | | Sarpogrelate + aspirin  (n=133) | | Total  (n=270) | |
| --- | --- | --- | --- | --- | --- | --- |
|  | n | % | n | % | n | % |
| Cardiac disorders | 2 | 1.5 | 6 | 4.5 | 8 | 3.0 |
| Congestive heart failure | 1 | 0.7 | 0 | 0.0 | 1 | 0.4 |
| Coronary atherosclerosis | 0 | 0.0 | 1 | 0.8 | 1 | 0.4 |
| Coronary disease | 0 | 0.0 | 1 | 0.8 | 1 | 0.4 |
| Ischemic cardiomyopathy | 1 | 0.7 | 0 | 0.0 | 1 | 0.4 |
| Myocardial infarction | 0 | 0.0 | 1 | 0.8 | 1 | 0.4 |
| Non ST segment elevation myocardial infarction | 0 | 0.0 | 1 | 0.8 | 1 | 0.4 |
| Paroxysmal atrial fibrillation | 0 | 0.0 | 1 | 0.8 | 1 | 0.4 |
| Sick sinus syndrome | 0 | 0.0 | 1 | 0.8 | 1 | 0.4 |
| Eye disorders | 0 | 0.0 | 2 | 1.5 | 2 | 0.7 |
| Diabetic retinopathy | 0 | 0.0 | 1 | 0.8 | 1 | 0.4 |
| Vitreous hemorrhage | 0 | 0.0 | 1 | 0.8 | 1 | 0.4 |
| Gastrointestinal disorders | 5 | 3.6 | 5 | 3.8 | 10 | 3.7 |
| Anal hemorrhage | 1 | 0.7 | 0 | 0.0 | 1 | 0.4 |
| Bowel perforation | 0 | 0.0 | 1 | 0.8 | 1 | 0.4 |
| Constipation | 1 | 0.7 | 1 | 0.8 | 2 | 0.7 |
| Gastric erosions | 1 | 0.7 | 0 | 0.0 | 1 | 0.4 |
| Gastritis | 1 | 0.7 | 1 | 0.8 | 2 | 0.7 |
| Ileus | 0 | 0.0 | 1 | 0.8 | 1 | 0.4 |
| Indirect inguinal hernia | 1 | 0.7 | 0 | 0.0 | 1 | 0.4 |
| Intestinal obstruction | 0 | 0.0 | 1 | 0.8 | 1 | 0.4 |
| General disorders and administration site conditions | 9 | 6.6 | 5 | 3.8 | 14 | 5.2 |
| Ankle edema | 1 | 0.7 | 0 | 0.0 | 1 | 0.4 |
| Application site purpura | 0 | 0.0 | 1 | 0.8 | 1 | 0.4 |
| Arterial stent occlusion | 0 | 0.0 | 1 | 0.8 | 1 | 0.4 |
| Chest pain | 0 | 0.0 | 1 | 0.8 | 1 | 0.4 |
| Death | 1 | 0.7 | 0 | 0.0 | 1 | 0.4 |
| Foot edema | 0 | 0.0 | 1 | 0.8 | 1 | 0.4 |
| Generalized aching | 1 | 0.7 | 0 | 0.0 | 1 | 0.4 |
| In-stent arterial stenosis | 1 | 0.7 | 0 | 0.0 | 1 | 0.4 |
| Leg edema | 2 | 1.5 | 0 | 0.0 | 2 | 0.7 |
| Puncture site hematoma | 1 | 0.7 | 0 | 0.0 | 1 | 0.4 |
| Unilateral leg swelling | 1 | 0.7 | 1 | 0.8 | 2 | 0.7 |
| Weakness generalized | 1 | 0.7 | 0 | 0.0 | 1 | 0.4 |
| Infections and infestations | 3 | 2.2 | 6 | 4.5 | 9 | 3.3 |
| Cellulitis | 1 | 0.7 | 0 | 0.0 | 1 | 0.4 |
| Cellulitis of toe | 1 | 0.7 | 0 | 0.0 | 1 | 0.4 |
| Cold | 0 | 0.0 | 1 | 0.8 | 1 | 0.4 |
| Gangrene toe | 0 | 0.0 | 1 | 0.8 | 1 | 0.4 |
| Mycobacterium avium intracellulare infection | 0 | 0.0 | 1 | 0.8 | 1 | 0.4 |
| Pneumonia | 0 | 0.0 | 3 | 2.3 | 3 | 1.1 |
| Septic shock | 1 | 0.7 | 0 | 0.0 | 1 | 0.4 |
| Injury, poisoning and procedural complications | 5 | 3.6 | 3 | 2.3 | 8 | 3.0 |
| Bruise | 1 | 0.7 | 0 | 0.0 | 1 | 0.4 |
| Bruising of leg | 1 | 0.7 | 0 | 0.0 | 1 | 0.4 |
| Burn local | 0 | 0.0 | 1 | 0.8 | 1 | 0.4 |
| Restenosis | 3 | 2.2 | 2 | 1.5 | 5 | 1.9 |
| Investigations | 1 | 0.7 | 7 | 5.3 | 8 | 3.0 |
| Alanine aminotransferase increased | 0 | 0.0 | 4 | 3.0 | 4 | 1.5 |
| Aspartate aminotransferase increased | 0 | 0.0 | 1 | 0.8 | 1 | 0.4 |
| Creatinine increased | 0 | 0.0 | 1 | 0.8 | 1 | 0.4 |
| Hemoglobin decreased | 1 | 0.7 | 0 | 0.0 | 1 | 0.4 |
| TSH increase | 0 | 0.0 | 1 | 0.8 | 1 | 0.4 |
| Metabolism and nutrition disorders | 2 | 1.5 | 2 | 1.5 | 4 | 1.5 |
| Gout | 0 | 0.0 | 1 | 0.8 | 1 | 0.4 |
| Gout attack | 0 | 0.0 | 1 | 0.8 | 1 | 0.4 |
| Hyperglycemia | 1 | 0.7 | 0 | 0.0 | 1 | 0.4 |
| Hyperlipidemia | 1 | 0.7 | 0 | 0.0 | 1 | 0.4 |
| Musculoskeletal and connective tissue disorders | 5 | 3.6 | 18 | 13.5 | 23 | 8.5 |
| Ankle swelling | 0 | 0.0 | 1 | 0.8 | 1 | 0.4 |
| Back pain | 0 | 0.0 | 1 | 0.8 | 1 | 0.4 |
| Calf pain | 1 | 0.7 | 2 | 1.5 | 3 | 1.1 |
| Cramps in legs | 0 | 0.0 | 1 | 0.8 | 1 | 0.4 |
| Foot discomfort | 0 | 0.0 | 1 | 0.8 | 1 | 0.4 |
| Joint pain | 0 | 0.0 | 1 | 0.8 | 1 | 0.4 |
| Knee pain | 1 | 0.7 | 3 | 2.3 | 4 | 1.5 |
| Leg cramps | 1 | 0.7 | 0 | 0.0 | 1 | 0.4 |
| Leg pain | 0 | 0.0 | 2 | 1.5 | 2 | 0.7 |
| Lumbar spinal stenosis | 1 | 0.7 | 0 | 0.0 | 1 | 0.4 |
| Muscle cramp | 1 | 0.7 | 0 | 0.0 | 1 | 0.4 |
| Pain ankle | 0 | 0.0 | 2 | 1.5 | 2 | 0.7 |
| Sacral pain | 0 | 0.0 | 1 | 0.8 | 1 | 0.4 |
| Spinal stenosis | 0 | 0.0 | 1 | 0.8 | 1 | 0.4 |
| Spondylolisthesis | 0 | 0.0 | 1 | 0.8 | 1 | 0.4 |
| Swelling of R knee | 0 | 0.0 | 1 | 0.8 | 1 | 0.4 |
| Neoplasms benign, malignant and unspecified (incl cysts and polyps) | 0 | 0.0 | 1 | 0.8 | 1 | 0.4 |
| Hepatocellular carcinoma | 0 | 0.0 | 1 | 0.8 | 1 | 0.4 |
| Nervous system disorders | 3 | 2.2 | 4 | 3.0 | 7 | 2.6 |
| Burning sensation | 0 | 0.0 | 1 | 0.8 | 1 | 0.4 |
| Cerebral haemorrhage | 1 | 0.7 | 0 | 0.0 | 1 | 0.4 |
| Cerebral infarction | 0 | 0.0 | 1 | 0.8 | 1 | 0.4 |
| Diabetic polyneuropathy | 0 | 0.0 | 1 | 0.8 | 1 | 0.4 |
| Ischemic stroke | 1 | 0.7 | 0 | 0.0 | 1 | 0.4 |
| Peripheral neuropathy | 1 | 0.7 | 0 | 0.0 | 1 | 0.4 |
| Tingling sensation | 0 | 0.0 | 1 | 0.8 | 1 | 0.4 |
| Psychiatric disorders | 0 | 0.0 | 2 | 1.5 | 2 | 0.7 |
| Insomnia | 0 | 0.0 | 2 | 1.5 | 2 | 0.7 |
| Renal and urinary disorders | 3 | 2.2 | 2 | 1.5 | 5 | 1.9 |
| Azotemia | 1 | 0.7 | 0 | 0.0 | 1 | 0.4 |
| Chronic kidney disease | 1 | 0.7 | 0 | 0.0 | 1 | 0.4 |
| Hematuria | 0 | 0.0 | 1 | 0.8 | 1 | 0.4 |
| Nocturia | 0 | 0.0 | 1 | 0.8 | 1 | 0.4 |
| Oliguria | 1 | 0.7 | 0 | 0.0 | 1 | 0.4 |
| Reproductive system and breast disorders | 1 | 0.7 | 1 | 0.8 | 2 | 0.7 |
| Benign prostatic hyperplasia | 1 | 0.7 | 1 | 0.8 | 2 | 0.7 |
| Respiratory, thoracic and mediastinal disorders | 6 | 4.4 | 0 | 0.0 | 6 | 2.2 |
| Chronic obstructive pulmonary disease | 1 | 0.7 | 0 | 0.0 | 1 | 0.4 |
| Dyspnea | 1 | 0.7 | 0 | 0.0 | 1 | 0.4 |
| Epistaxis | 2 | 1.5 | 0 | 0.0 | 2 | 0.7 |
| Pulmonary edema | 2 | 1.5 | 0 | 0.0 | 2 | 0.7 |
| Skin and subcutaneous tissue disorders | 2 | 1.5 | 2 | 1.5 | 4 | 1.5 |
| Diabetic foot | 0 | 0.0 | 1 | 0.8 | 1 | 0.4 |
| Ecchymosis | 0 | 0.0 | 1 | 0.8 | 1 | 0.4 |
| Localised itching | 1 | 0.7 | 0 | 0.0 | 1 | 0.4 |
| Petechia | 1 | 0.7 | 0 | 0.0 | 1 | 0.4 |
| Surgical and medical procedures | 3 | 2.2 | 4 | 3.0 | 7 | 2.6 |
| Below knee amputation | 0 | 0.0 | 2 | 1.5 | 2 | 0.7 |
| Disarticulation | 0 | 0.0 | 1 | 0.8 | 1 | 0.4 |
| Kidney transplant | 1 | 0.7 | 0 | 0.0 | 1 | 0.4 |
| Toe amputation | 1 | 0.7 | 0 | 0.0 | 1 | 0.4 |
| Wound debridement | 1 | 0.7 | 1 | 0.8 | 2 | 0.7 |
| Vascular disorders | 6 | 4.4 | 6 | 4.5 | 12 | 4.4 |
| Claudication | 3 | 2.2 | 0 | 0.0 | 3 | 1.1 |
| Coldness of lower extremities | 0 | 0.0 | 1 | 0.8 | 1 | 0.4 |
| Hematoma | 0 | 0.0 | 2 | 1.5 | 2 | 0.7 |
| Lymphocele | 0 | 0.0 | 1 | 0.8 | 1 | 0.4 |
| Popliteal arterial stenosis | 1 | 0.7 | 0 | 0.0 | 1 | 0.4 |
| Superficial femoral artery occlusion | 1 | 0.7 | 0 | 0.0 | 1 | 0.4 |
| Tibial artery occlusion | 1 | 0.7 | 1 | 0.8 | 2 | 0.7 |
| Tibial artery stenosis | 0 | 0.0 | 1 | 0.8 | 1 | 0.4 |

**Table S2. Univariate and multivariate analyses for restenosis**

|  | Univariate | | Multivariate | |
| --- | --- | --- | --- | --- |
|  | OR (95% CI) | p-value | OR (95% CI) | p-value |
| treatment group |  |  |  |  |
| aspirin+clopidogrel | 1 (ref) |  | 1 (ref) |  |
| aspirin+sarpogrelate | 0.633 (0.299-1.344) | 0.2340 | 0.269 (0.045-1.622) | 0.1522 |
| age, y | 1.000 (0.961-1.041) | 0.9981 | 0.967 (0.897-1.042) | 0.3804 |
| sex |  |  |  |  |
| female | 1 (ref) |  | 1 (ref) |  |
| male | 0.439 (0.168-1.152) | 0.0945 | 0.230 (0.011-4.840) | 0.3447 |
| diabetes mellitus |  |  |  |  |
| no | 1 (ref) |  | 1 (ref) |  |
| yes | 1.701 (0.767-3.772) | 0.1911 | 0.382 (0.055-2.656) | 0.3308 |
| current smoking |  |  |  |  |
| no | 1 (ref) |  | 1 (ref) |  |
| yes | 0.600 (0.264-1.363) | 0.2225 | 1.160 (0.208-6.463) | 0.8652 |
| coronary artery disease |  |  |  |  |
| no | 1 (ref) |  | 1 (ref) |  |
| yes | 1.197 (0.500-2.865) | 0.6870 | 0.499 (0.024-10.510) | 0.6551 |
| statin treatment |  |  |  |  |
| no | 1 (ref) |  | 1 (ref) |  |
| yes | 0.988 (0.468-2.082) | 0.9740 | 0.496 (0.069-3.556) | 0.4850 |
| antiplatelet treatment |  |  |  |  |
| no | 1 (ref) |  | 1 (ref) |  |
| yes | 0.901 (0.422-1.920) | 0.7864 | 0.788 (0.123-5.064) | 0.8016 |
| hemodialysis |  |  |  |  |
| no | 1 (ref) |  | 1 (ref) |  |
| yes | 2.000 (0.597-6.698) | 0.2610 | 2.708 (0.068-107.145) | 0.5956 |
| body mass index, kg/m^2^ | 0.997 (0.897-1.108) | 0.9495 | 0.983 (0.740-1.305) | 0.9046 |
| cholesterol, mg/dl | 1.001 (0.992-1.010) | 0.9132 | 0.994 (0.970-1.018) | 0.6204 |
| Rutherford classification |  |  |  |  |
| 2-3 | 1 (ref) |  | 1 (ref) |  |
| 4-6 | 0.997 (0.375-2.652) | 0.9956 | 0.242 (0.011-5.479) | 0.3731 |
| TASC Ⅱ-A classification |  |  |  |  |
| A-B | 1 (ref) |  | 1 (ref) |  |
| C-D | 1.853 (0.585-5.868) | 0.2945 | 0.445 (0.035-5.612) | 0.5311 |
| TASC Ⅱ-F classification |  |  |  |  |
| A-B | 1 (ref) |  | 1 (ref) |  |
| C-D | 1.560 (0.726-3.354) | 0.2545 | 23.095 (1.536-347.336) | 0.0232 |
| BTK runoff |  |  |  |  |
| 0-1 | 1 (ref) |  | 1 (ref) |  |
| 2-3 | 0.965 (0.368-2.534) | 0.9428 | 0.945 (0.076-11.743) | 0.9650 |
| concomittant procedure |  |  |  |  |
| no | 1 (ref) |  | 1 (ref) |  |
| inflow | 0.739 (0.237-2.309) | 0.6033 | 2.137 (0.238-19.199) | 0.4979 |
| outflow | 1.294 (0.505-3.305) | 0.5917 | 0.448 (0.033-6.082) | 0.5459 |
| intervention type |  |  |  |  |
| balloon | 1 (ref) |  | 1 (ref) |  |
| stent | 0.355 (0.160-0.787) | 0.0107 | 0.020 (0.001-0.330) | 0.0062 |

**Figure S1. Non-inferiority test for the primary outcome of target lesion restenosis (Full analysis set)**


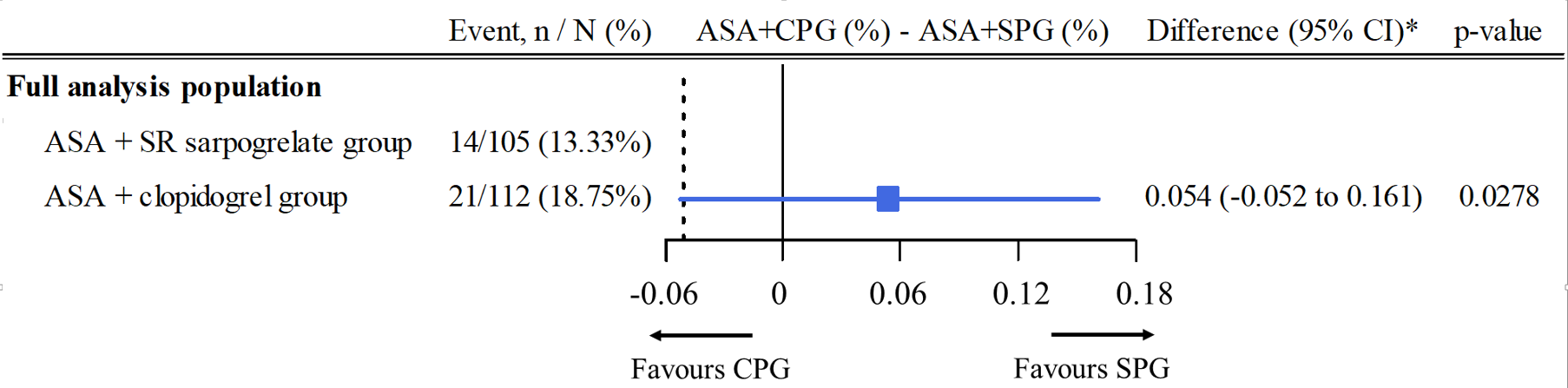

Supplement: Supplementary file 1 — Supplementary Information. [file 41598_2023_29006_MOESM1_ESM.docx]
